# Germicide Fenaminosulf Promots Gall Formation of *Zizania latifolia* without directly affecting the growth of endophytic fungus *Ustilago esculenta*

**DOI:** 10.1186/s12870-022-03803-6

**Published:** 2022-08-30

**Authors:** Fang Li, Juefeng Zhang, Haiying Zhong, Jianming Chen

**Affiliations:** Institute of Plant Protection and Microbiology, Zhejiang Academy of Agricultural Sciences, Hangzhou, 310021 China

**Keywords:** *Zizania latifolia*, Fungal endophyte, Gall formation, Fenaminosulf, RNA sequencing

## Abstract

*Zizania latifolia* is a popular aquatic vegetable in China because of its enlarged edible stems resulting from persistent infection by a fungal endophyte, *Ustilago esculenta*. Fenaminosulf (FM) is a germicide that can be used to improve agricultural crop yields. In *Z. latifolia* fields, appropriate spraying of FM not just controls diseases, but also promotes an earlier harvest of *Z. latifolia*. In this study, we show that the timing of gall formation was advanced and the plant’s yield was increased significantly under a high concentration treatment of FM. Yet FM had a strong inhibitory effect on the growth of *U. esculenta in vitro*, while the transcript levels of mating-type alleles, cell metabolism-related genes and chitin synthase genes were all substantially downregulated. Through a transcriptome analysis, we investigated changes in gene expression of the host *Z. latifolia* and fungal endophyte *U. esculenta* in response to FM. FM directly affected the growth of *Z. latifolia* by altering the expression level of genes involved in plant-pathogen interactions, plant hormone signal transduction and some metabolism pathways. By contrast, FM had little effect on *U. esculenta* growing inside of *Z. latifolia*. Collectively, our results provide a more in-depth understanding of the molecular processes that promote gall formation in *Z. latifolia*, while also identifying potential targets for genetic manipulation to improve the yield and quality of *Z. latifolia*, in a safer and more effective way.

**Supplementary Information:**

The online version contains supplementary material available at 10.1186/s12870-022-03803-6.

## Introduction

*Zizania latifolia* (Griseb.) Turcz. ex Stapf, belonging to the Oryzeae of the Gramineae, is an asexual aquatic vegetable with an edible swelling structure which forms gall that has been cultivated in Asia for more than 2000 years [1–5]. It is cultivated at scattered locations throughout Korea and Japan, and in China it is widely cultivated from northern Beijing to southern Guangdong province, and from eastern Sichuan province to Shanghai. The largest area under *Z. latifolia* cultivation area in China is found in Jiangsu and Zhejiang provinces. It is estimated that more than 20,000 ha are under cultivation with this crop plant in Zhejiang province alone and that 500,000 tons of fresh product are harvested from this plant species each year [1, 6]. In addition to its great economic value, it also has good edible value. The harvested edible gall of *Z. latifolia* is plump and tender, with little fiber, high protein, and abundant antioxidant properties that may prevent hypertension and cardiovascular disease [7–9], improve insulin resistance [10] and have anti-obesity effects [11]. According to photosensitivity and harvesting time of this species, more than 100 local varieties may be divided into two main ecotypes of *Z. latifolia* [12]. One ecotype is a single-season plant, harvested once per year in the fall (from August to November) after planting in spring. Another is a double-season crop, also planted in the spring, but harvested twice: in fall (hereon, ‘Autumn-Jiao’) and the following summer (‘Summer-Jiao’). Temperature and illumination are the key factors influencing *Z. latifolia* growth [1, 12].

The basidiomycetes fungus *Ustilago esculenta* could specifically colonize *Z. latifolia* hosts by invading their meristems. There, the mycelium of *U. esculenta* grows in or between the cells in the meristem near the stem tip, which prevents the infected plant from flowering and causes the formation of the localized, edible swelling structure [13–17]. The plant pathogen *U. esculenta* is unique among the smut fungi that are plant pathogens, which mainly parasitize grasses and cause ‘smut disease’ [18–21], because it does not induce chlorosis symptoms in the host, or necrosis, but instead induces the formation of differentiated galls on the culm upon infection [15, 22–26]. In China, *Z. latifolia* is commonly referred to as “*Jiaobai*” and categorized into three types according to the interaction mode with *U. esculenta*, normal *Jiaobai*, grey *Jiaobai*, and male *Jiaobai* [27, 28]. The normal *Jiaobai* form edible galls at the base region of the plant and are full of fungal hyphae due to infecting with mycelial strains of *U. esculenta*. The grey *Jiaobai* is filled with dark-colored teliospores due to infecting with sporifial strains of *U. esculenta* that could not form normal mycelial morphology similar to that in normal *Jiaobai*. The grey *Jiaoba* is often discarded due to their unacceptable taste and potential for triggering hypersensitivity pneumonitis when consumed [16, 25, 26]. The male *Jiaobai* without *U. esculenta* infection fails to produce any galls and will exhibit normal flowering [1, 23]. Some research has speculated that different phenotypes of normal and male *Jiaobai* in the fields arose from differentiated strains of *U. esculenta*, which spurred diverse host defense responses [28]. In sum, *U. esculenta* infection is a critical reason for normal development of *Jiaobai.* The genetic interactions between *U. esculenta* and the host plant were recently revealed by whole-genome sequencing and comparative transcriptome analyses [28–32].

Fenaminosulf (FM, p-dimethylaminobenzenediazosodium sulfonate) is a common protective germicide, with special effects on the diseases caused by *Pythium* and *Aphanomyces* that could afflict vegetable, cotton, tobacco, rice, and wheat crops [33–35]. It can also be served as a very effective seed and soil treatment agent that is able to control a variety of seed and soil borne diseases [36, 37]. FM is characterized by a strong absorption and penetration, such that it is readily absorbed by root and leaf surfaces. However, FM stimulates adverse effects when on human skin and it causes harm to the aquatic environment. Appropriate spraying of FM not only controls diseases in *Z. latifolia* fields but it also advances the harvest of the *Z. latifolia* crop. Currently, in some *Z. latifolia*-producing areas in China, the edible gall was harvested by spraying FM during the growth of *Z. latifolia*. Yet the lack of a scientific and rationally application method could cause FM to affect the growth and safety of *Z. latifolia* crops, run against the farmers’ interests, and even pollute the environment. So far, the mechanism by which FM acts upon *Z. latifolia* and *U. esculenta* has not been fully elucidated. We investigated the effects of FM on the process of gall formation, and carried out intensive research of molecular processes that could promote gall formation of *Z. latifolia*. The findings are beneficial for the further exploration and exploitation the mechanism of gall formation for improving the yield and quality of *Z. latifolia* in a safer and more efficient way.

In recent years, FM has been widely used for promoting the formation of the gall in fields of *Z. latifolia*, but in the process its harvest timing has been significantly affected. In this report, besides the formation time of gall, the yield and regulatory mechanism of *Z. latifolia* were monitored under different concentrations of applied FM. In parallel, the growth of *U. esculenta* under the action of FM was analyzed in the laboratory. In addition, transcriptome analysis was performed to search for and elucidate FM’s mechanistic action on *Z. latifolia* and *U. esculenta*. Our results not only provide a comprehensive description about the effect of FM on *Z. latifolia* and *U. esculenta* respectively, but also lay the theoretical basis for the mechanism of gall formation in *Z. latifolia.*

## Materials and methods

### Strains and growth conditions

The *Z. latifolia* plants (cultivar ‘Longjiao 2’) used in this study were planted in Yuhang District, Hangzhou City, Zhejiang Province. Longjiao 2 was one of the most widely cultivated double-season *Jiaobai* varieties in eastern China. The galls were first harvested in autumn, at 100–120 days after planting in spring, and then harvested in the following summer. The Summer-Jiao of Longjiao 2 had a higher yield, so it was used for the experiments. The *U. esculenta* strain was isolated from sporulating galls of grey *Jiaobai* cultured at 28 °C on YEPS medium (1% yeast extract, 2% peptone, 2% sucrose).

### Field experiment and investigation

Longjiao 2 was cultivated in five plots, each with 16 clumps of *Jiaobai*. The five plots were divided into four experimental groups treated with different concentrations of FM (Yuelian Chemical Co., Ltd., Shanghai, China) and a control group without any FM treatment. The experimental groups 1–4 were treated three times with a concentration of 0.45 g/L, 0.9 g/L, 1.8 g/L, and 3.6 g/L of FM respectively. The interval of each application was 8 days. Fertilizer and water management, as well as pest control practices, were kept the same across the five plots throughout the experiment. The experiment lasted from February to June 2018.

According to local harvesting habits, the size of gall was significantly enlarged and 1–2 cm white flesh of pseudostem was exposed, indicating that *Z. latifolia* was ripe and needed to be harvested immediately. Beginning with the formation of gall, the date and yield of each harvest were recorded. Galls were collected for the following experiments.

### FM stress tolerance of *U. esculenta*

Triplicate assays were carried out to assess the possible effects on the tolerance of FM in sporidia of *U. esculenta* isolated from grey *Jiaobai* (Longjiao 2). The sporidia suspension was collected from a colony of teliospores grown at 28 °C on the YEPS solid medium. Aliquots of 5-μl sporidia suspension were centrally spotted on the plates (90 mm diameter) of YEPS supplemented with the gradient concentrations of FM (0.009, 0.018, 0.027 and 0.036 g/L). After a 10-day incubation at 28 °C, colony diameters were cross-measured and relative growth rates calculated as the ratios of specifically stressed colony areas to those in blank controls (not including any agent).

The trend in colony growth (*y*) of each strain over the intensity (*x*) of a given stress level was fitted to the equation *y* = 1/[1 + exp.(a + b*x*)], yielding parameters for estimating the effective concentration of FM to suppress 50% viability (EC_50_).

Temporal transcript patterns of some selected genes (Table S1) in the RNA extracts of sporidia YEPS cultures during the 10-day incubation at 28 °C were determined via qRT-PCR. All total RNAs were extracted from the stressed (0.018 g/L FM) and unstressed cultures (without FM) using RNAiso Plus (Takara, Japan), from which 5-ug RNA samples were transcribed using a PrimeScriptTM RT reagent kit (Takara, Japan). A dilution (10 ng/μl) of the synthesized cDNA was then used as a template for qRT-PCR with paired primers (Supplementary Table 1) for each gene. The fungal β-actin rRNA served as internal standard. Each qRT-PCR assay included three samples as replicates. The relative transcript level of a given gene was calculated as the ratio of its transcript in the stressed culture over that in the unstressed control [38].

### RNA extraction, cDNA library construction, and RNA-Seq

*Jiaobai* samples were selected from the control group and the 3.6 g/L FM group in the experimental field plots. The total RNA of *Z. latifolia*, and that of *U. esculenta*, were extracted from the mature galls at the same development stage, by the TRIzol Reagent (Invitrogen, USA) and following the manufacturer’s instructions. All samples were treated with DNase I (New England Biolabs, MA, USA). The purity and quantity of total RNA was assessed with a NanoPhotometer® spectrophotometer (IMPLEN, CA, USA) and a Qubit® 2.0 Flurometer (Life Technologies, CA, USA), respectively. RNA integrity was confirmed using the Agilent Bioanalyzer 2100 system (Agilent Technologies, CA, USA). The total high-quality RNA isolated from three independent biological replicates of *Jiaobai* samples were used individually to construct cDNA libraries.

The RNA-Seq of two samples, namely control group and 3.6 g/L FM stressed group, each with three biological replicate, was performed on the Illumina Hiseq 2000 platform from which 150-bp paired-end reads were generated. Raw sequences were deposited in the NCBI Short Read Archive (SRA) database (http://www.ncbi.nlm.nih.gov/Traces/sra/). The accession number of RNA-seq data was PRJNA 669494 for *Z. latifolia* and PRJNA 669466 for *U. esculenta*. Raw reads in the FASTQ format were first filtered, by removing any reads containing adapter sequences and the low-quality reads. At the same time, the Q20, Q30, GC-content, and sequence duplication level of the clean data were each calculated. Cleaned reads containing both host and *U. esculenta* sequences were mapped onto the *Z. latifolia* reference genome [30] and *U. esculenta* reference genome [32], using the Bowtie2 software tool [39].

### Transcriptome annotation, expression profiling, data analysis and data validation

Gene function was annotated by homology searches against the NCBI non-redundant protein (Nr) database, NCBI nucleotide (Nt) database, Swiss-Prot protein database, euKaryotic Orthologous Groups (KOG), Kyoto Encyclopedia of Genes and Genomes (KEGG), Gene Ontology (GO), and the Protein family (Pfam) database.

Expression levels of genes were measured using counts of reads normalized by their respective lengths, in the Cufflinks 2.0.2 package under its default settings (http://cole-trap-nell-lab.github.io/cufflinks/) for normalization (genometric), followed by their distribution analysis in terms of Fragments Per Kilobase of exon per Million mapped reads (FPKM) units. The different expression genes (DEG) seq package was used to identify those DEGs between control group and 3.6 g/L FM stressed group; it provides statistical routines for determining differential expression levels in digital gene expression data by applying a model based on the negative binomial distribution. Resulting p-values were adjusted with the Benjamini and Hochberg’s approach for controlling the false discovery rate (FDR). Genes with an adjusted p-value <0.05 as detected by DEGSeq were designated as differentially expressed. The fold-changes for a given gene’s expression between samples were calculated as log2 (treatment FPKM value/control FPKM value).

The DEGs related signal pathways were analyzed in *Z. latifolia* compared with those in *Zea mays* by KEGG [40–42]. Related maps were also obtained from KEGG.

To confirm the results of transcriptome comparisons, qRT-PCR was performed on 16 randomly selected genes in *Z. latifolia* and *U. esculenta*. The samples were consistent with those used by the transcriptome. The primers for qRT-PCR were listed in Supplementary Table S2. The experiment and data calculation method of qRT-PCR were consistent with those mentioned above.

## Results

### FM affects the gall formation and yield of *Z. latifolia*

**Fig. 1:**
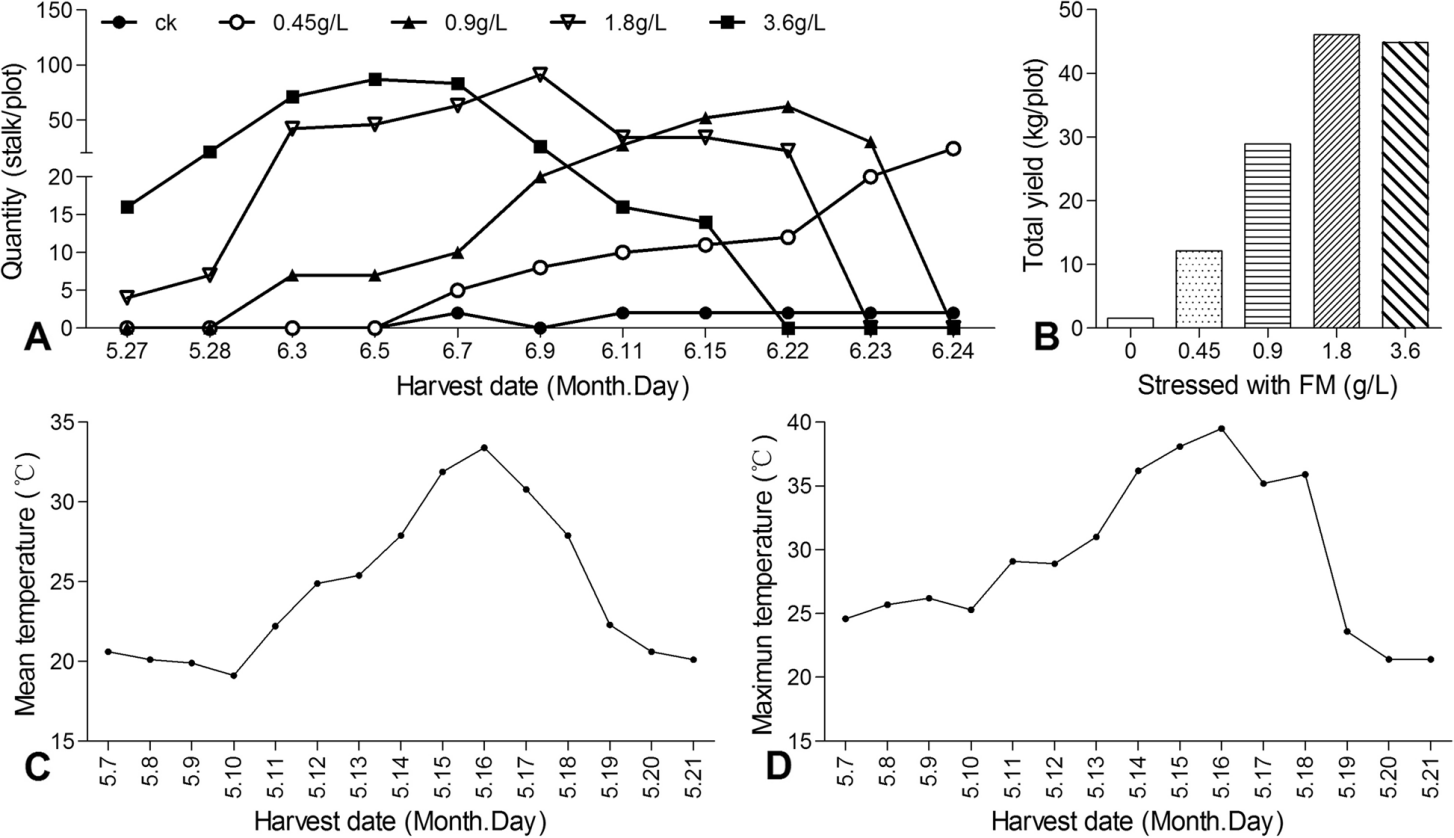
Effects of fenaminosulf (FM) on the gall formation and yield of *Zizania latifolia*. **A** Daily yield of *Z. latifolia* recorded after incubation in the presence of different concentrations of FM. The stems were collected from May 27 to June 24 from each plot treated with 0, 0.45, 0.9, 1.8, and 3.6 g/L FM. **B** The total yield of *Z. latifolia* under different concentrations of FM. The total weight of *Z. latifolia* in each plot treated with 0, 0.45, 0.9, 1.8, and 3.6 g/L FM was calculated. **C** and **D**. Mean and maximum temperatures during the gall formation period in the field experiment. The mean and maximum temperatures of each day during the harvest period from May 7 to 21 were obtained from local meteorological bureau

After treatment with different concentrations of FM, the timing of gall formation and yield of *Z. latifolia* was greatly affected. As Fig. 1A shows, the *Z. latifolia* began harvest from May 27 onward. Under the influence of 3.6 g/L FM, the harvest time of *Z. latifolia* was markedly advanced. The peak harvest period of *Z. latifolia* under the 1.8, 0.9, and 0.45 g/L FM treatments was respectively delayed by 4, 17, and 19 days, when compared with that under the 3.6 g/L FM application. The control group maintained a lower harvest volume during the entire harvest period.

The FM concentration applied not only affected the timing of gall formation, but also their total yield from *Z. latifolia* (Fig. 1B). When treated with a higher concentration of FM, a higher yield of *Z. latifolia* was harvestable. Under the 1.8 g/L and 3.6 g/L FM treatments, the yields of *Z. latifolia* respectively were 46.07 kg and 44.86 kg, which corresponded to 0.60-fold and 0.55-fold increases over the 0.9 g/L FM treatment (28.88 kg). Moreover, when compared with the yield of the 0.45 g/L treatment group (12.09 kg), their increments were 2.8-fold and 2.7-fold greater, respectively.

The harvest timing of *Z. latifolia* was the latest in the control group, for which it never reached the peak period during the whole harvest period (Fig. 1A, B). Accordingly, the control group had the lowest yield, at only 1.61 kg. Since fertilizer and water management and pest control were identical among the treatment groups, weather conditions were monitored (Fig. 1C, D). Around May 7, the *Z. latifolia* entered the period of gall formation, about 20 days before the onset of the harvest period (From May 7 to 27). According to the information provided by the local meteorological bureau, from May 7 to 12, the average temperature was around 20 °C, a suitable temperature for gall formation. After May 17, however, the experimental field experienced high-temperature weather exceeding 30 °C whose daily average temperature was above 25 °C for 6 days. This spell of high temperature happened to occur in the critical period of gall formation in the control group.

### FM affects the sporidia growth of *U. esculenta in vitro*

**Fig. 2:**
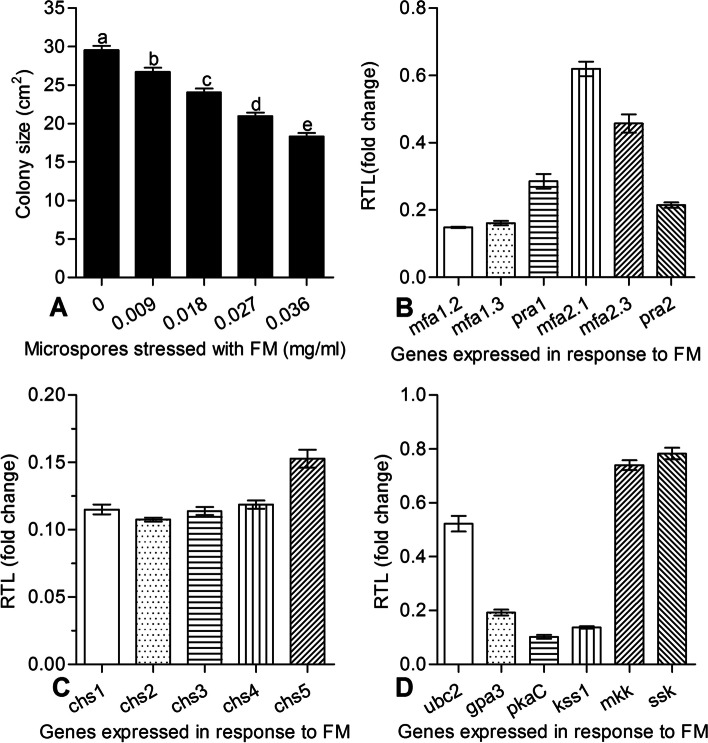
Effects of fenaminosulf (FM) on the growth of *Ustilago esculenta*. **A** Growth of *U. esculenta* on stressed media. The sporidia of *U. esculenta* isolated from grey *Jiaobai* were grown on PDA with gradient concentrations of FM (0.009, 0.018, 0.027 and 0.036 g/L). Colony sizes were calculated after 10-day growth at 28 °C. Different letters above the column bars indicate significant differences (*p* < 0.05). Error bars: SD of the mean from three replicate assays. **B**–**D**. Relative transcript levels of selected stress-responsive genes in the stressed cultures of *U. esculenta* (versus the control). The cultures were stressed with 0.018 g/L FM during a 10-day incubation at 28 °C and assayed via qRT-PCR with paired primers in the Table S1. Error bars: SD of the mean from three cDNA samples

A high concentration of FM completely inhibited sporidia growth of *U. esculenta* in the YEPS culture. The sporidia began growing so long as the concentration was below 0.09 g/L; corresponding inhibition rates of 0.009 g/L, 0.018 g/L, 0.027 g/L, and 0.036 g/L FM on sporidia growth were 9.5, 18.6, 29, and 37.8%, respectively (Fig. 2A). Hence, the inhibition rate increased with a rising concentration of FM. The FM resistance was quantified as EC_50_ after exposure to FM culture_._ The EC_50_ was 0.042 g/L.

Seventeen relative genes possibly involved in the altered responses of sporidia to FM (0.018 g/L) versus control group were assessed for their transcript levels via qRT-PCR with paired primers (Table S1). Sporidia isolated from teliospores could be divided into two strains: one containing *mfa*1.2, *mfa*1.3, and *pra*1 alleles, while the other contained *mfa*2.1, *mfa*2.3, and *pra*2 alleles that were α mating-type alleles in *U. esculenta*. The expression levels of all of them were significantly repressed, by 38.1–85.2% (Fig. 2B). The drastic repression of mating relative genes indicated that FM impaired the conjugation formation, the initial step of the mating process in *U. esculenta*.

Under the stress of FM (0.018 g/L), the expression levels of cell metabolism-related genes were also significantly repressed (by 21.8–89.8%) in *U. esculenta* (Fig. 2C). Remarkably, *gpa3*, *kss1,* and *pkaC* of *U. esculenta* were all downregulated more than 80%. Among five chitin synthase genes (*chs1* through *chs5*) responding to FM (Fig. 2D), all were largely downregulated by 84.7–89.2%. Transcriptional levels of these genes related to cell metabolism and cell wall disturbing were all repressed in response to FM; this strongly implied FM could considerably affect the normal metabolic growth of *U. esculenta*.

### Genome-wide expression analysis of FM effects upon *Z. latifolia* and *U. esculenta*

To determine how FM affects the gall formation in *Z. latifolia* and the interaction between it and *U. esculenta*, a transcriptome analysis was performed. The galls harvest from the control group (without FM) and experimental group receiving the 3.6 g/L FM treatment were used to collect transcriptional information for *Z. latifolia* and *U. esculenta*. Samples with three replicates were prepared from these two groups.

**Fig. 3:**
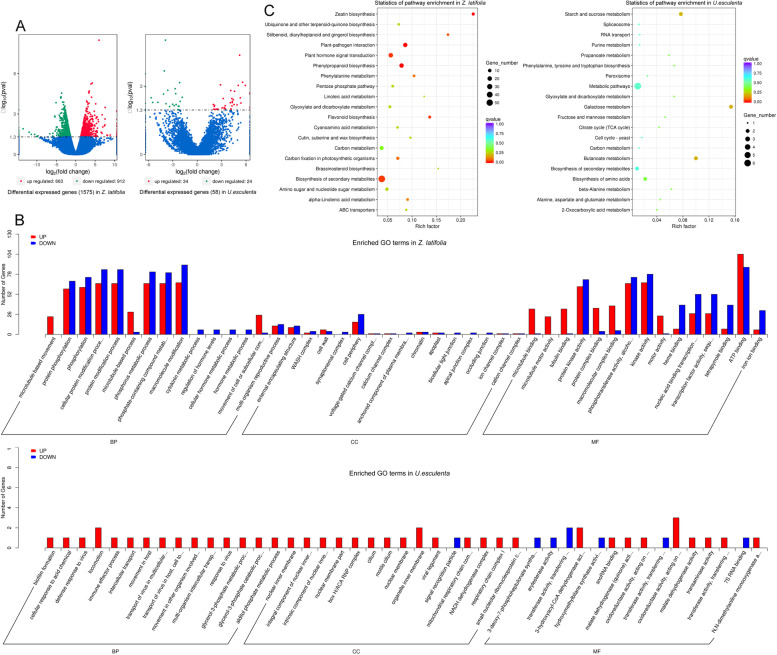
Overview of RNA-Seq data. **A** Volcano-plot of differentially expressed genes (DEGs) from the stressed group compared with the control in *Zizania latifolia* and *Ustilago esculenta*, respectively. Blue- and red-colored splashes represent significantly upregulated and downregulated genes. Blue-colored splashes represent genes without significant differences in their expression levels. **B** Gene ontology (GO) classification of DEGs between stressed culms and the control in *Z. latifolia* and *U. esculenta*, respectively. Along the horizontal axis is the enriched GO term, and on the vertical axis is the number of DEGs in a given term. Red- and blue-colored bars represent upregulated and downregulated genes, respectively. **C** Kyoto Encyclopedia of genes and Genomes (KEGG) pathway enrichment analysis of DEGs in *Z. latifolia* and *U. esculenta*, respectively. The ordinate represents the pathway name, the abscissa represents the rich factor, and the point size represents the number of DEGs in that pathway, while the point color denotes the differing Q-value ranges

Deep RNA sequencing produced the 9.2 × 10^7^ and 8.6 × 10^7^ valid reads for the *Z. latifolia*, 2.4 × 10^7^ and 2.4 × 10^7^ valid reads for the *U. esculenta* libraries, in the control and experimental group, respectively. These sequence reads were mapped onto the genome of *Z. latifolia* and *U. esculenta*, resulting in the identification of 46,092 and 7347 genes derived respectively from the *Z. latifolia* and *U. esculenta* libraries. Comparative analysis between the control and experimental groups’ expression profiles revealed 663 and 912 transcripts upregulated and downregulated in *Z. latifolia*, and 34 and 24 transcripts upregulated and downregulated in *U. esculenta*, respectively (Fig. 3A). To validate the gene changes identified by comparative transcriptomic analysis, we randomly selected 16 genes in *Z. latifolia* and *U. esculenta* for qRT-PCR. As shown in Fig. S1, all genes behaved similarly between qRT-PCR and RNA-seq data.

GO enrichment analyses were used to annotate the function of differentially expressed genes (DEGs), which could be assigned to three major categories: molecular function (MF), biological process (BP), and cellular component (CC). In *Z. latifolia*, the unigenes were categorized into 45 GO terms, with most DEGs belonging to BP and MF (Fig. 3B). In the BP category, phosphorylation, protein modification, phosphorus- and phosphate-containing compound metabolic process, and macromolecule modification were the top four classes enriched by regulated transcripts. In the MF category, many genes were categorized as protein kinases and binding proteins. In *U. esculenta*, the number of DEGs was small (Table S4), and there was an average distribution in these three enriched GO categories.

As Fig. 3C shows, KEGG pathway enrichment analysis revealed a set of genes involved in galactose metabolism, butanoate metabolism, and starch and sucrose metabolism that were differentially expressed as enriched (corrected *p*-value <0.2) in *U. esculenta*. In *Z. latifolia*, a fair number of DEGs (corrected *p*-value <0.05) involved in plant–pathogen interaction, phenylpropanoid biosynthesis, zeatin biosynthesis, plant hormone signal transduction, flavonoid biosynthesis, biosynthesis of secondary metabolites, and biosynthesis of stilbenoid, diarylheptanoid and gingerol were evidently enriched. Generally, those genes with a corrected *p*-value <0.05 could be considered as an enriched item.

**Fig. 4:**
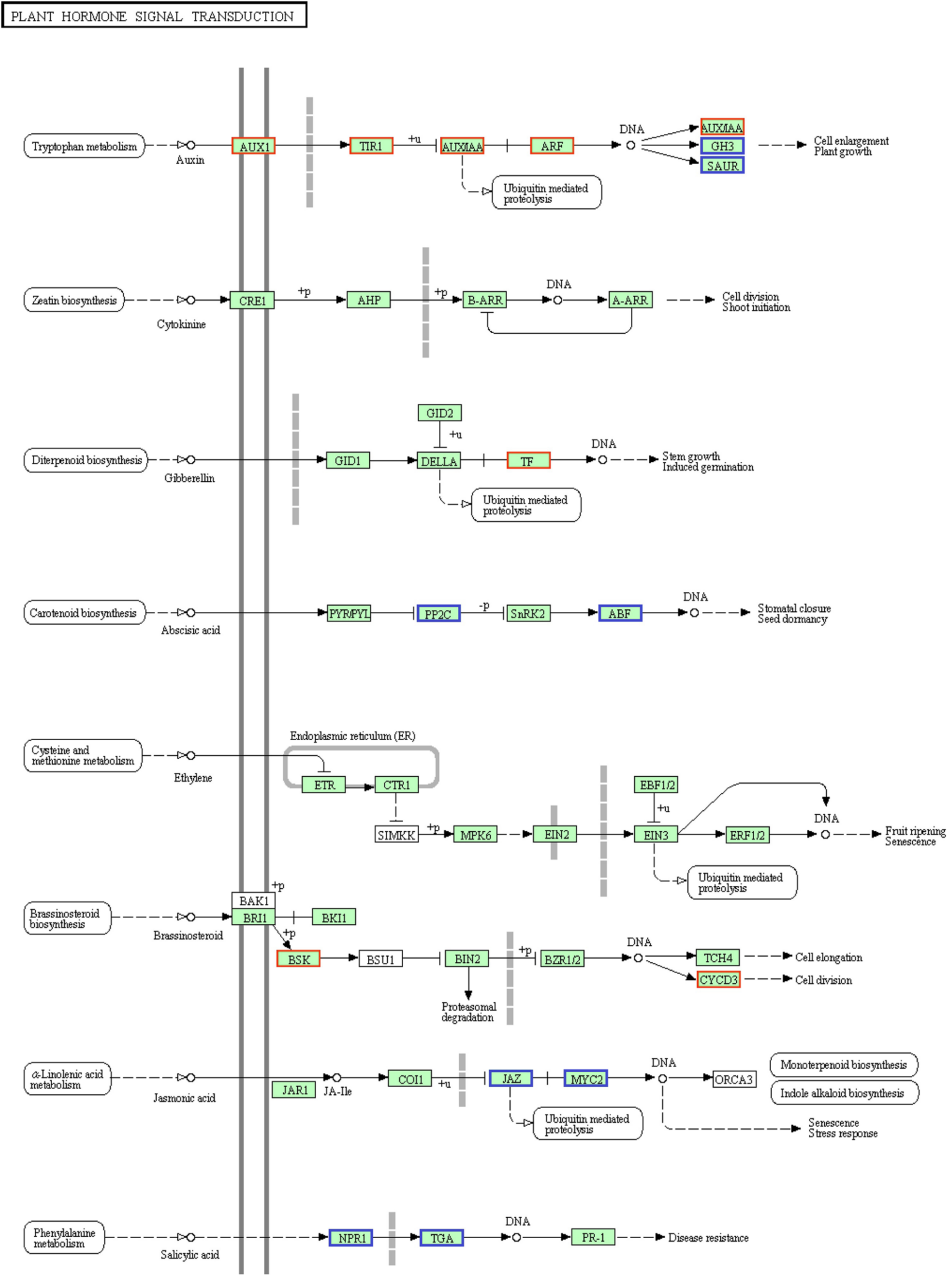
Gene expression effects of fenaminosulf on plant-pathogen interactions in *Zizania latifolia*. Genes downregulated (boxed in blue) or upregulated (boxed in red) in the stressed culm group versus control. The map came from KEGG and map number was zma04626

**Fig. 5:**
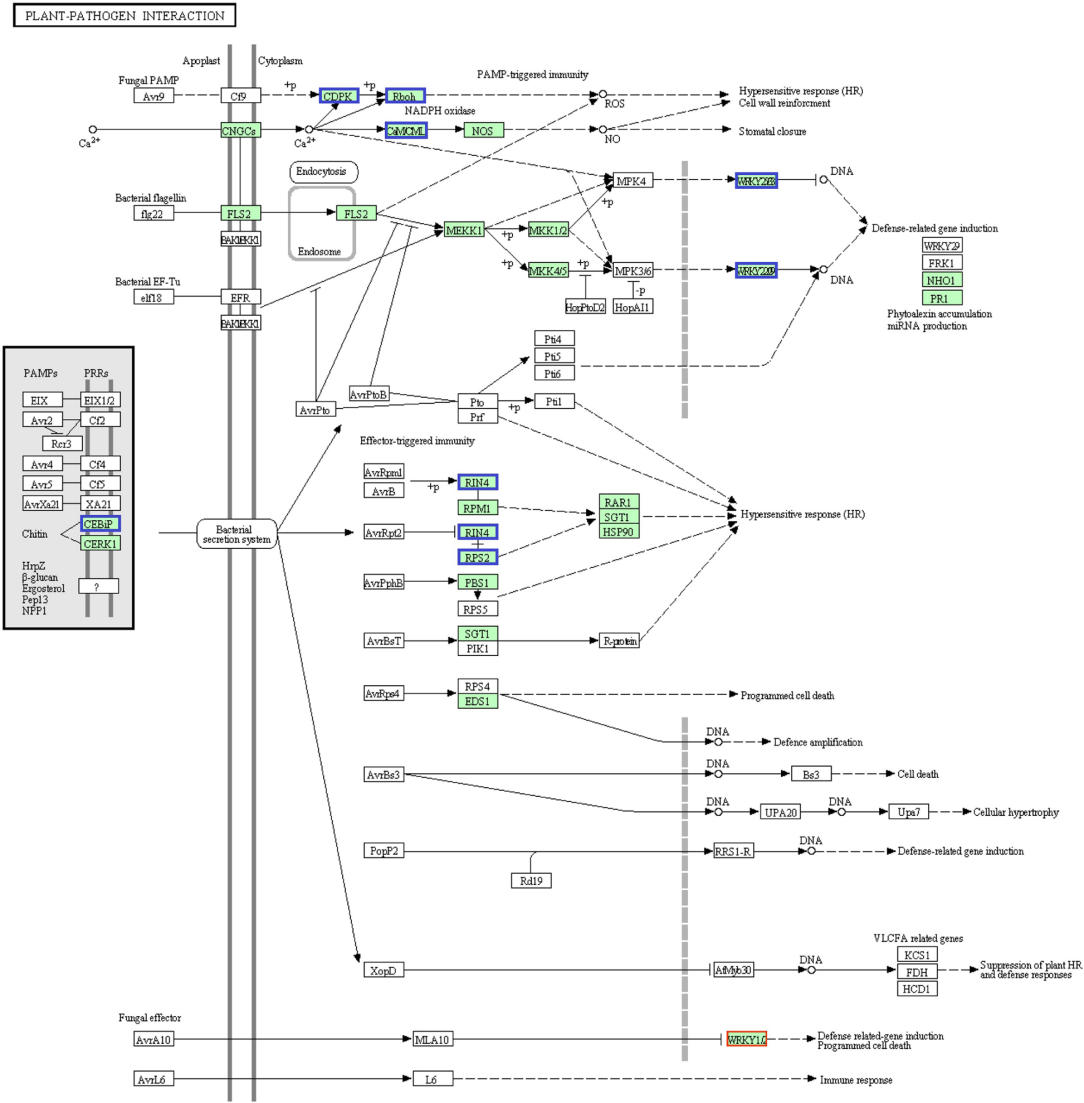
Gene expression effects of fenaminosulf on plant hormone signal transduction in *Zizania latifolia*. Genes downregulated (boxed in blue) or upregulated (boxed in red) in the stressed culm group versus the control. The map came from KEGG and map number was zma04075

In *Z. latifolia*, the transcript levels of 77.7% of the DEGs (101 of 130) in the enrichment items were downregulated under the stress of FM (Table S3). Specifically, 94.4% (17 of 18), 73.7% (14 of 19), 83.3% (5 of 6), and 76.8% (43 of 56) of DEGs were downregulated dramatically in the process of plant-pathogen interaction (see also Fig. 4), phenylpropanoid biosynthesis, flavonoid biosynthesis, and biosynthesis of secondary metabolites. Notably, in the process of zeatin biosynthesis (7 genes) and stilbenoid, diarylheptanoid, and gingerol biosynthesis (4 genes), all of the DEGS were especially downregulated. For the process of plant hormone signal transduction, 55% (11 of 20) of the involved DEGs were downregulated (Table S3; see also Fig. 5) and those were related to auxin signaling, seed dormancy, stress response, and disease resistance.

A previous study identified 170 and 205 putative host genes, along with 53 and 71 *U. esculenta* genes involved in the initial and late stage of gall formation [31]. Compared with those genes, we found that 6 of 13 DEGs in *Z. latifolia* under FM stress had the same trend of change, in that they were all downregulated (Table 1). In *U. esculenta*, however, no DEGs with the same change pattern could be discerned by comparison in our experiment.

**Table 1:** Gene expression results for the full list of overlapping transcripts between paired libraries, comparing (i) differentially expressed genes (DEGs) in fenaminosulf-stressed *Zizania latifolia*, and (ii) DEGs involved in the initial triggering and swelling stage of gall formation in *Z. latifolia* ^a^

Locus tag	Annotation	log2[foldchange] (i)	log2[foldchange] (ii)^a^
Zlat_10004561	Calcium-binding protein CML31	−1.5199	−14.0995
Zlat_10006578	Disease resistance protein 2 RPS2	−1.8883	−10.2046
Zlat_10001910	Peroxidase 52 PER52	2.1065	−8.611
Zlat_10008844	Cationic peroxidase SPC4	−1.4769	3.4341
Zlat_10001161	Cytokinin dehydrogenase 4 CKX4	−1.9672	−9.4798
Zlat_10003321	Adenylate isopentenyltransferase 1 IPT1	−1.6601	1.2887
Zlat_10026402	Gibberellin 2-beta-dioxygenase 8	−2.081	−9.0526
Zlat_10019656	4-hydroxyphenylacetaldehyde oxime monooxygenase	−2.4564	−10.676
Zlat_10020835	1-aminocyclopropane-1-carboxylate oxidase	−2.4359	−7.6176
Zlat_10006128	Isocitrate lyase	−100	6.7474

^a^These data come from the research of Wang [31]

## Discussion

The plant *Z. latifolia* forms a shuttle-like gall with a unique flavor, making it a widely cultivated aquatic vegetable in China. But under certain unfavorable conditions, such as high-dose radiation, water deficiency, unsuitable fungicide application, adverse growing temperatures, or if plants are infected with the sporifial strain (forming the grey *Jiaobai*) or escape from fungal infection (forming the male *Jiaobai*) [25, 26, 43], the yield of *Z. latifolia* may be substantially reduced.

As a common fungicide in *Z. latifolia* fields, FM is able to not only effectively inhibit disease but also promote the gall formation of *Z. latifolia*. Although the time of gall formation and yield of *Z. latifolia* in the control group were markedly affected by high temperature, in the experimental group they were significantly advanced and increased under the effect of FM and most pronounced under the high concentration treatment. The peak harvest period of *Z. latifolia* was 19 days sooner under the 3.6 g/L FM treatment than the 0.45 g/L FM one. When treated with 1.8 g/L and 3.6 g/L FM, the yields of *Z. latifolia* were significantly increased when compared with the 0.45 g/L FM treatment. It seems reasonable to suggest that FM could significantly promote the gall formation of *Z. latifolia*, leading to an earlier harvest peak and a higher yield.

The gall formation of *Z. latifolia* was affected not only by the external environment but also by the internal symbiotic smut. Previous studies had reported that *U. esculenta* gene expression contributes to gall formation in *Z. latifolia* [31, 43]. Therefore, the use of fungicides in the field was done very cautiously to prevent potential negative impacts on the growth of *U. esculenta*, which would threaten the gall formation of its host, *Z. latifolia*. Here, the effect of FM on the growth of *U. esculenta* isolated from grey *Jiaobai* plants was tested *in vitro*. The sporidia of *U. esculenta* were incapable of growing under the action of high concentrations of FM, beginning to do so only when its concentration fell below 0.09 g/L. Clearly, the FM chemical compound has a strong inhibitory effect on *U. esculenta* growth. Under the stress of FM, the transcript levels of mating-type alleles, cell metabolism-related genes, and chitin synthase genes in *U. esculenta* were all repressed considerably. This suggests FM impaired the initial mating action and normal growth of *U. esculenta*, and interpretation that is in line with the effectiveness of FM as a fungicide.

Through the transcriptome analysis, we investigated changes in gene expression of the host *Z. latifolia* and the pathogen *U. esculenta* in response to FM, finding several noteworthy differences between control and stressed group. In *Z. latifolia*, functional analysis of DEGs revealed that most of them related to phosphorylation, protein modification, macromolecule modification, phosphorus and phosphate-containing compound metabolic process, protein kinases, and binding proteins. In *U. esculenta*, the number of DEGs was small, and these genes were evenly distributed in three enriched GO terms categories.

The plant–pathogen interaction between *Z. latifolia* and *U. esculenta* is unique because *U. esculenta* induces the formation of differentiated galls upon infection of its host *Z. latifolia* [15]*.* Unlike *Ustilago maydis*, which causes the production of large tumors on all aerial organs in maize plants [20], *U. esculenta* induces gall formation in a more moderated way, by only inducing culm hypertrophy without obvious symptoms of tissue damage in other parts of its host [25, 26]. *Z. latifolia* has a strong response to the infection of *U. esculenta*, although the pathogen has the opportunity to survive in its plant tissues [31]. Here we focused on changes of expressed genes in the processes of plant–pathogen interaction and plant hormone signal transduction by transcriptomic analysis, finding that FM elicited a plant response reaction to stimulus, thus implying a critical role of FM in promoting gall formations of *Z. latifolia*. Most of the identified DEGs exhibited decreased expression in the process of this plant-pathogen interaction. Compared with those genes involved in the swelling stage of gall formation, FM enhanced the expressions of two genes encoding the calcium-binding protein CML31 and disease resistance protein RPS2, which participate in plant response to external stimuli. Applying FM also influenced the expression of other genes, mainly those associated with inhibition, suggesting a stressed plant provides *U. esculenta* with superior opportunities to penetrate and expand in its plant tissues.

The induction of galls in host plants by microbes is undoubtedly dependent on the activity of plant hormones, particularly elevated levels of cytokinins and auxins, which trigger a signaling cascade leading to activation of mitotic cell division [20, 22, 44]. In our study, the transcript levels of genes encoding AUX1 (auxin influx carrier), TIR1 (transport inhibitor response 1-like protein), AUX/IAA (auxin-responsive protein IAA), and ARF (auxin response factor 23) were elevated, in contrast to genes encoding GH3 (Indole-3-acetic acid-amido synthetase) and SAUR (auxin-induced protein) that were downregulated. This revealed that in a stressed plant, transcript levels involved in auxin signaling prophase were all upregulated, and upon reaching the prophase, the transcript levels of auxin signaling-related proteins underwent differing changes. This may arise from the need to maintain an adequate auxin balance in *Z. latifolia*. Furthermore, the expression levels of genes encoding BSK and CYCD3, which were related to cell division and phytochrome-interacting factor TF known to promote stem growth and induce germination, were upregulated. The expression levels of other genes involved in seed dormancy, stress response, and disease resistance were all downregulated. The expression level changes of these genes were closely associated with gall formation occurring sooner in host plants. All the changed gene expression levels seem converged towards advancing the onset of gall formation of *Z. latifolia*.

Secondary metabolites, such as lignin synthesized through the phenylpropanoid biosynthesis pathway, can promote plant resistance to pathogenic infection [45]. In our study, most of the DEGs in this pathway, as well as some enzyme genes in other metabolic pathways, showed diminished expression in the stressed plant group, suggesting these stressed plants provide a less resistant way for the penetration and expansion of *U. esculenta* in their plant tissues.

Under the stress of 3.6 g/L FM, the transcriptome of the pathogen *U. esculenta* in host *Z. latifolia* was also analyzed. The number of fungal DEGs found was very small, however, at only 58 genes. There were 20 most enriched pathway terms in *U. esculenta* and these were mainly concentrated in metabolism pathways. By way of comparison with genes involved in gall formation, the DEGs of *U. esculenta* were not directly related to gall formation, revealing that FM did not promote gall formation of *Z. latifolia* by affecting the growth of *U. esculenta*, but rather by directly affect *Z. latifolia* plants.

Under field conditions, FM played a key role in promoting gall formation of *Z. latifolia*, leading to an earlier peak in harvesting and increased production. Although FM significantly inhibited the growth of *U. esculenta* isolated from *Z. latifolia*, it actually had little effect on *U. esculenta* when it grows within *Z. latifolia* hosts. From our investigation of genome-wide transcriptome profiles and expression profiles of *Z. latifolia*, we inferred that FM could directly affect the growth of *Z. latifolia* by altering gene expression level involved in plant-pathogen interactions, plant hormone signal transduction, and some metabolism pathway. Besides their possible association with gall formation under FM stress, how the candidate genes function in terms of inducing gall formation remains unknown. The effects of FM on *Z. latifolia* could bring huge economic benefits to farmers, but the safety of its use and the residue it leaves must be seriously considered. Some studies reported that FM could induce a wide range of damage to DNA in *Allium. cepa* root nuclei [35] and it was slightly toxic to *Lepomis macrochirus* and *Oncorhynchus mykiss* [46, 47]. Although the concentration of FM used in our field experiment was relatively low, and FM easily disintegrates under sunlight, we recommend that it is still should be used carefully. This will spur us to find a safer way to promote gall formation of *Z. latifolia*. Collectively, the findings in this study deepen our understanding of molecular processes underlying the promotion of gall formation in *Z. latifolia*. They could prove timely and useful in further exploratory research and for exploiting the gall formation genes to improve the yield and quality of *Z. latifolia*a in way that is both safer and more efficient.

## Conclusions

In summary, we conclude that FM has a positive, promoting effect on accelerating gall formation, advancing the peak time of harvest and increasing yield of *Z. latifolia* under field conditions. Although *U. esculenta* is essential for gall formation, the growth of fungi is slightly affected in the process of promoting gall formation under FM stress, suggesting that FM does not impair gall formation through affecting the growth of *U. esculenta* directly. Comparative transcriptomic analysis indicates that FM could directly affect the growth of *Z. latifolia* by altering the expression levels of key genes involved in plant-pathogen interactions, plant hormone signal transduction, and some metabolism pathways. For safety issues, we strongly recommend using FM with extreme caution, though FM application could significantly promot gall formation of *Z. latifolia* and consequently bring huge economic benefits. This study gives more comprehensive insights into mechanisms promoting gall formation in *Z. latifolia* under FM treatment and provides clues to develop new efficient strategies for promoting gall formation and improving the yield of *Z. latifolia*.

## Supplementary Information

**Additional file 1: Table S1.** Paired primers used for qRT-PCR in *Ustilago esculenta*. **Table S2.** Paired primers used in *Zizania latifolia* and *Ustilago esculenta* for confirming data validation of transcriptome by qRT-PCR. **Table S3.** List of differentially expressed genes in *Zizania latifolia* under fenaminosulf stress. **Table S4.** List of differentially expressed genes in *Ustilago esculenta* under fenaminosulf stress. **Figure S1.** Comparison of gene expression patterns obtained by RNA-Seq and qRT-PCR.

## Data Availability

The datasets generated for this study are available upon request to the corresponding author. Raw sequences were deposited in the NCBI Short Read Archive (SRA) database (http://www.ncbi.nlm.nih.gov/Traces/sra/). The accession number of RNA-seq data was PRJNA 669494 for *Z. latifolia* and PRJNA 669466 for *U. esculenta*.
