# Supplementary material for: Germicide Fenaminosulf Promots Gall Formation of Zizania latifolia without directly affecting the growth of endophytic fungus Ustilago esculenta
Source: BMC Plant Biol. 2022 Aug 30;22:418. doi: 10.1186/s12870-022-03803-6 (PMC9426258; doi:10.1186/s12870-022-03803-6)
Supplement: Supplementary file 1 — Additional file 1: Table S1. Paired primers used for qRT-PCR in Ustilago esculenta. Table S2. Paired primers used in Zizania latifolia and Ustilago esculenta for confirming data validation of transcriptome by qRT-PCR. Table S3. List of differentially expressed genes in Zizania latifolia under fenaminosulf stress. Table S4. List of differentially expressed genes in Ustilago esculenta under fenaminosulf stress. Figure S1. Comparison of gene expression patterns obtained by RNA-Seq and qRT-PCR. [file 12870_2022_3803_MOESM1_ESM.docx]

**Germicide Fenaminosulf Promots Gall Formation of *Zizania latifolia* without directly affecting the growth of endophytic fungus *Ustilago esculenta***

**Fang Li^1^, Juefeng Zhang^1^, Haiying Zhong^1^, Jianming Chen^1^***

^1^ State Key Laboratory for Managing Biotic and Chemical Threats to the Quality and Safety of Agro-products, Hangzhou, 310021, China; Institute of Plant Protection and Microbiology, Zhejiang Academy of Agricultural Sciences, Hangzhou, 310021, China

**^*^ Correspondence:**

Jianming Chen

chenjm63@163.com

**Supplementary tables**

**Table S1** Paired primers used for qRT-PCR in *Ustilago esculenta*.

| **Primers** | **Paired sequences (5′-3′) *** | **Purpose** |
| --- | --- | --- |
| qβ-actin-F/ R | CAATGGTTCGGGAATGTGC/ GGGATACTTGAGCGTGAGGA | Detecting of *β-actin* transcript |
| q*mfa*1.2-F/ R | TGTTCTCCATCTTCACTCA/ CGACAATACATGTGGAGTAG | Detecting of *mfa*1.2 transcript |
| q*mfa*1.3-F/R | GGACGCTCTTACTCTCTT/ GCAACGATACATCCAGAA | Detecting of *mfa*1.3 transcript |
| q*pra*1-F/R | CGTTATGTCAGGCTTCTC/ AGATGTAGGCGGATAGAG | Detecting of *pra*1 transcript |
| q*mfa*2.1-F/R | TCACTATCTTCGAGACTGTT/ AGTAGTTGGCAAGCTGTC | Detecting of *mfa*2.1 transcript |
| q*mfa*2.3-F/R | TCGCCATTTTCTCTTTCTCG/ ATGATGCAACCGCTAGA | Detecting of *mfa*2.3 transcript |
| q*pra*2-F/R | ATAGGCTTGTGCTGTAGT/ CGACGAAGAATAACGGAAT | Detecting of *pra*2 transcript |
| q*ubc*2-F/R | TGGAGGAAGACGACGAAAGCT/ CATGCTCATGGACTACCAGAGT | Detecting of *ubc*2 transcript |
| q*gpa*3-F/R | TGGGTAATTGCCTTTCTTCGAC/ TGGATGATCTTCATCTGCTTGA | Detecting of *gpa*3 transcript |
| q*pkaC*-F/R | CATGAAGGAGGTCAAGGT/ GCACATACACATCCAAGG | Detecting of *pkaC* transcript |
| q*kss*1-F/R | AATCCACGCAACATTCTC/ ATCTTCTTGATGGCTACCT | Detecting of *kss*1 transcript |
| q*mkk*-F/R | CCATTCCATCAGCATCAG/ CAAGTCGTGTTGTTGTAATC | Detecting of *mkk* transcript |
| q*ssk*-F/R | ACACATCTCGCAAGTAAG/ GGCTTCCTTCTGGTATTG | Detecting of *ssk* transcript |
| q*chs*1-F/R | TCGTATCCTCCTCACATC/ CATCATATCGTCATCGTCAT | Detecting of *chs*1 transcript |
| q*chs*2-F/R | GCCGACCTACATTATCATC/ TCTTGACTTCCACCATCT | Detecting of *chs*2 transcript |
| q*chs*3-F/R | GGTGGTGTATGTGTTGTG/ CGGTATCGTTGTGGAATC | Detecting of *chs*3 transcript |
| q*chs*4-F/R | ACTGCCTACTCTGAATCTT/ GTCTCGTTGTCGTAATCG | Detecting of *chs*4 transcript |
| q*chs*5-F/R | TACATCTCACACCACCTC/ ACCAGCGTAGTCAAGTAT | Detecting of *chs*5 transcript |

**Table S2** Paired primers used in *Zizania latifolia* and *Ustilago esculenta* for confirming data validation of transcriptome by qRT-PCR.

| **Gene name/id** | **Paired sequences (5′-3′) *** | **Target** |
| --- | --- | --- |
| β-actin-F/ R | GACGGTGAGGATATCAAGCC/ GCGAGGGCAACCGACAATAC | in *Zizania latifolia* |
| Zlat_10006578 | CGAGAAGTATGCTACAAGA/ CACACTCCTTAACAATGATG | in *Zizania latifolia* |
| Zlat_10008844 | CTTTGGACTGTCACCTTC/ CCATCTCGTCAAGAATCTC | in *Zizania latifolia* |
| Zlat_10048065 | CAACTCCTACCGATTCTC/ AATGGCTGTATTCCTCTC | in *Zizania latifolia* |
| Zlat_10041370 | GACGAGGAGATGAAGGAG/ CATCATGCACTTGAACTC | in *Zizania latifolia* |
| Zlat_10001910 | TCGTCAAGATGGGCAACA / TTAGTTGGGCTTCCTGCA | in *Zizania latifolia* |
| Zlat_10047787 | ACACCTTTACTCACATTCC/ CACATCCACCTTGTATCG | in *Zizania latifolia* |
| Zlat_10017452 | TGAGTTCGTCTTCTTCTTCTT / TCACCAGTTCACCACCTA | in *Zizania latifolia* |
| Zlat_10000572 | GCTACAACGGATTATTCG / CATCACCAACAAGCATTC | in *Zizania latifolia* |
| β-actin-F/ R | CAATGGTTCGGGAATGTGC/ GGGATACTTGAGCGTGAGGA | in *Ustilago esculenta* |
| g813 | GAGTATTCGCAGGTTGTT / GTGGTTCTGATGCTTGTA | in *Ustilago esculenta* |
| g3269 | GGAACCGTGTATGTCTAC / AGTAGTTGATGCTGTTGAC | in *Ustilago esculenta* |
| g2660 | GTATCAAGAAGACCGAGTA / CGATCATGTTGGAGTAGT | in *Ustilago esculenta* |
| g980 | CAACAGAGATGAGTCCAA / GATGTAAGGTCAGGAAGG | in *Ustilago esculenta* |
| g5895 | TGCTGTGATCTACAATGG / GATGATGACGACTTGGAA | in *Ustilago esculenta* |
| g352 | CTACCAAGAAGCAGAACA / TGTCGGTGTCTACTGATA | in *Ustilago esculenta* |
| g2684 | CAACTATGCCTCTACATCC / CGACTTTGACTGTTTCTTG | in *Ustilago esculenta* |
| g6523 | CGCTCTTGGAGTAATCTT / CGTGGTGGTAGTATTGAA | in *Ustilago esculenta* |

**Table S3** List of differentially expressed genes in *Zizania latifolia* under fenaminosulf stress.

| Locus tag | Annotation | log2FoldChange |
| --- | --- | --- |
| KEGG:zma04626 Plant-pathogen interaction  Sample number/Background number:18/209 Corrected P-value:0.000788571 | | |
| Zlat_10034518 | WRKY transcription factor 2 WRKY2 | 1.8221 |
| Zlat_10027326 | WRKY transcription factor 22 WRK22 | -2.296 |
| Zlat_10006341 | WRKY transcription factor 27 WRK27 | -2.5964 |
| Zlat_10028790 | WRKY transcription factor 33 WRK33 | -2.1091 |
| Zlat_10017671 | WRKY transcription factor 33 WRK33 | -2.0059 |
| Zlat_10004543 | Calcium-dependent protein kinase 14 CDPK14 | -3.4139 |
| Zlat_10028613 | Calcium-dependent protein kinase 30 CDPK30 | -1.7203 |
| Zlat_10008743 | Calcium-binding protein CML14 | -2.0117 |
| Zlat_10041370 | Calcium-binding protein CML24 | -2.6476 |
| Zlat_10006767 | Calcium-binding protein CML27 | -1.7111 |
| Zlat_10004561 | Calcium-binding protein CML31 | -1.5199 |
| Zlat_10006578 | Disease resistance protein 2 RPS2 | -1.8883 |
| Zlat_10030262 | Chitin elicitor-binding protein | -1.7397 |
| Zlat_10048179 | Respiratory burst oxidase homolog protein C | -1.8535 |
| Zlat_10026801 | Respiratory burst oxidase homolog protein E | -1.7885 |
| Zlat_10019842 | RPM1-interacting protein 4 RIN4 | -1.4583 |
| Zlat_10038158 | PTI1-like tyrosine-protein kinase 3 PTI13 | -1.925 |
| Zlat_10036946 | -//- | -1.8764 |
| KEGG:zma00940 Phenylpropanoid biosynthesis  Sample number/Background number:19/242 Corrected P-value: 0.000788571 | | |
| Zlat_10041351 | Cationic peroxidase 1 PER1 | -3.1259 |
| Zlat_10036307 | Peroxidase 1 PER1 | 2.246 |
| Zlat_10007768 | Peroxidase 19 PER19 | 2.0968 |
| Zlat_10029728 | Peroxidase 47 PER47 | -12.011 |
| Zlat_10001910 | Peroxidase 52 PER52 | 2.1065 |
| Zlat_10046751 | Peroxidase 52 PER52 | 2.9818 |
| Zlat_10003203 | Peroxidase 72 PER72 | -2.7887 |
| Zlat_10007582 | Cinnamyl alcohol dehydrogenase 5 CADH5 | -2.262 |
| Zlat_10037056 | Cinnamyl alcohol dehydrogenase 6 CADH6 | -3.0534 |
| Zlat_10004185 | Cinnamyl alcohol dehydrogenase 9 CADH9 | -1.9164 |
| Zlat_10048065 | Probable inactive beta-glucosidase 14 BGL14 | -4.5313 |
| Zlat_10047787 | Beta-glucosidase 30 BGL30 | 8.1727 |
| Zlat_10045750 | Cytochrome P450 CYP73A100 | -2.0429 |
| Zlat_10045747 | Cytochrome P450 CYP73A100 | -2.6201 |
| Zlat_10015120 | Cinnamoyl-CoA reductase 1 CCR1 | -3.867 |
| Zlat_10035005 | 4-coumarate--CoA ligase 5 4CL5 | -1.6962 |
| Zlat_10016473 | Tricin synthase 1 | -1.9036 |
| Zlat_10008844 | Cationic peroxidase SPC4 | -1.4769 |
| Zlat_10045749 | Trans-cinnamate 4-monooxygenase | -2.7848 |
| KEGG:zma00908 Zeatin biosynthesis  Sample number/Background number:7/31 Corrected P-value: 0.000788571 | | |
| Zlat_10001161 | Cytokinin dehydrogenase 4 CKX4 | -1.9672 |
| Zlat_10032604 | Cytokinin dehydrogenase 5 CKX5 | -1.753 |
| Zlat_10029015 | Cytokinin dehydrogenase 5 CKX5 | -1.9567 |
| Zlat_10029016 | Cytokinin dehydrogenase 5 CKX5 | -2.1255 |
| Zlat_10003044 | Cytokinin dehydrogenase 11 CKX11 | -1.6667 |
| Zlat_10017743 | Cis-zeatin O-glucosyltransferase 1 CZOG1 | -2.8285 |
| Zlat_10003321 | Adenylate isopentenyltransferase 1 IPT1 | -1.6601 |
| KEGG:zma04075 Plant hormone signal transduction  Sample number/Background number:20/355 Corrected P-value: 0.018559854 | | |
| Zlat_10037646 | Indole-3-acetic acid-amido synthetase GH3.8 GH38 | -1.9171 |
| Zlat_10005384 | Serine/threonine-protein kinase At4g35230 | 1.8145 |
| Zlat_10007749 | serine/threonine-protein kinase At4g35230 | 2.4567 |
| Zlat_10005792 | Auxin response factor 11 ARF11 | 7.5341 |
| Zlat_10005787 | Auxin-induced protein X10A AX10A | -3.8671 |
| Zlat_10015482 | Auxin transporter-like protein 2 LAX2 | 2.1862 |
| Zlat_10017452 | Transcription factor APG | 1.6209 |
| Zlat_10009390 | Transcription factor MYC2 | -1.596 |
| Zlat_10022833 | bZIP transcription factor TRAB1 | -1.4779 |
| Zlat_10040352 | Transport inhibitor response 1-like protein TIR1A | 2.0898 |
| Zlat_10035141 | TGACG-sequence-specific DNA-binding protein TGA-2.1 TGA21 | -2.5162 |
| Zlat_10022265 | protein phosphatase 2C 37 P2C37 | -1.6287 |
| Zlat_10026591 | Protein TIFY 3A TIF3A | -1.5511 |
| Zlat_10022690 | Regulatory protein NPR3 | -1.6198 |
| Zlat_10006293 | Protein TIFY 10A TI10A | -1.6425 |
| Zlat_10018497 | Cyclin-D3-2 CCD32 | 2.1198 |
| Zlat_10000572 | -//- | 3.6105 |
| Zlat_10026592 | -//- | -1.9869 |
| Zlat_10000259 | -//- | 2.5918 |
| KEGG:zma00941 Flavonoid biosynthesis  Sample number/Background number:6/44 Corrected P-value: 0.018934388 | | |
| Zlat_10045747 | Cytochrome P450 CYP73A100 | -2.6201 |
| Zlat_10045750 | Cytochrome P450 CYP73A100 | -2.0429 |
| Zlat_10045749 | Trans-cinnamate 4-monooxygenase | -2.7848 |
| Zlat_10016473 | Tricin synthase 1 | -1.9036 |
| Zlat_10001216 | Anthocyanidin reductase | -3.0914 |
| Zlat_10003508 | Chalcone--flavonone isomerase | 2.2501 |
| KEGG:zma01110 Biosynthesis of secondary metabolites  Sample number/Background number:56/1481 Corrected P-value: 0.022291996 | | |
| Zlat_10032403 | Serine hydroxymethyltransferase 7 GLYC7 | -2.1046 |
| Zlat_10041351 | Cationic peroxidase 1 PER1 | -3.1259 |
| Zlat_10036307 | Peroxidase 1 PER1 | 2.246 |
| Zlat_10007768 | Peroxidase 19 PER19 | 2.0968 |
| Zlat_10029728 | Peroxidase 47 PER47 | -12.011 |
| Zlat_10001910 | Peroxidase 52 PER52 | 2.1065 |
| Zlat_10046751 | Peroxidase 52 PER52 | 2.9818 |
| Zlat_10003203 | Peroxidase 72 PER72 | -2.7887 |
| Zlat_10008844 | Cationic peroxidase SPC4 | -1.4769 |
| Zlat_10041502 | Peroxisomal (S)-2-hydroxy-acid oxidase GLO3 | -1.8284 |
| Zlat_10007582 | Cinnamyl alcohol dehydrogenase 5 CADH5 | -2.262 |
| Zlat_10037056 | Cinnamyl alcohol dehydrogenase 6 CADH6 | -3.0534 |
| Zlat_10004185 | Cinnamyl alcohol dehydrogenase 9 CADH9 | -1.9164 |
| Zlat_10048065 | Probable inactive beta-glucosidase 14 BGL14 | -4.5313 |
| Zlat_10047787 | Beta-glucosidase 30 BGL30 | 8.1727 |
| Zlat_10045750 | Cytochrome P450 CYP73A100 | -2.0429 |
| Zlat_10045747 | Cytochrome P450 CYP73A100 | -2.6201 |
| Zlat_10034541 | Cytochrome P450 724B1 | 2.0021 |
| Zlat_10037791 | 3-ketoacyl-CoA synthase 5 KCS5 | -100 |
| Zlat_10018082 | 3-ketoacyl-CoA synthase 11 KCS11 | -4.2539 |
| Zlat_10009486 | 3-ketoacyl-CoA synthase 12 KCS12 | -2.4227 |
| Zlat_10015120 | Cinnamoyl-CoA reductase 1 CCR1 | -3.867 |
| Zlat_10035005 | 4-coumarate--CoA ligase 5 4CL5 | -1.6962 |
| Zlat_10026402 | Gibberellin 2-beta-dioxygenase 8 | -2.081 |
| Zlat_10032857 | Gibberellin 2-beta-dioxygenase | -1.7147 |
| Zlat_10031241 | Protein ECERIFERUM 1 CER1 | 1.7321 |
| Zlat_10026392 | Protein ECERIFERUM 1 CER1 | 8.8863 |
| Zlat_10017156 | Lipoxygenase 2.3 | -1.3266 |
| Zlat_10003050 | Lipoxygenase 5 LOX5 | -1.7654 |
| Zlat_10045844 | ATP-dependent 6-phosphofructokinase 3 PFKA3 | -1.9501 |
| Zlat_10046954 | Fructose-bisphosphate aldolase cytoplasmic isozyme | -1.77 |
| Zlat_10032926 | Fructose-1,6-bisphosphatase | -3.7715 |
| Zlat_10020097 | Fructose-1,6-bisphosphatase | -3.2867 |
| Zlat_10012970 | ATP-citrate synthase alpha chain protein 2 ACLA2 | 1.6908 |
| Zlat_10043163 | Non-specific phospholipase C4 NPC4 | -1.9603 |
| Zlat_10023644 | Glycerol-3-phosphate 2-O-acyltransferase 4 GPAT4 | -1.6075 |
| Zlat_10045749 | Trans-cinnamate 4-monooxygenase | -2.7848 |
| Zlat_10019656 | 4-hydroxyphenylacetaldehyde oxime monooxygenase | -2.4564 |
| Zlat_10045635 | 12-oxophytodienoate reductase 1 OPR1 | -1.7521 |
| Zlat_10016473 | Tricin synthase 1 | -1.9036 |
| Zlat_10039160 | Malate dehydrogenase | -2.0036 |
| Zlat_10020835 | 1-aminocyclopropane-1-carboxylate oxidase | -2.4359 |
| Zlat_10028912 | Solanesyl-diphosphate synthase 1 SPS1 | 1.6388 |
| Zlat_10003508 | Chalcone--flavonone isomerase | 2.2501 |
| Zlat_10030369 | Obtusifoliol 14-alpha demethylase | -2.0888 |
| Zlat_10001216 | Anthocyanidin reductase | -3.0914 |
| Zlat_10025055 | Branched-chain-amino-acid aminotransferase 3 BCAT3 | -1.3997 |
| Zlat_10012130 | Farnesyl pyrophosphate synthase | -2.3845 |
| Zlat_10011777 | Pyruvate kinase isozyme A KPYA | 1.7043 |
| Zlat_10018948 | Glycine cleavage system H protein | -2.2227 |
| Zlat_10020498 | Salicylate carboxymethyltransferase | -2.0801 |
| Zlat_10039130 | Allene oxide synthase 1 | -1.724 |
| Zlat_10006128 | Isocitrate lyase | 100 |
| Zlat_10003321 | Adenylate isopentenyltransferase 1 IPT1 | -1.6601 |
| Zlat_10000934 | -//- | -2.6327 |
| Zlat_10000435 | -//- | -1.8873 |
| KEGG:zma00945 Stilbenoid, diarylheptanoid and gingerol biosynthesis  Sample number/Background number:4/23 Corrected P-value: 0.039972715 | | |
| Zlat_10045747 | Cytochrome P450 CYP73A100 | -2.6201 |
| Zlat_10045750 | Cytochrome P450 CYP73A100 | -2.0429 |
| Zlat_10045749 | Trans-cinnamate 4-monooxygenase | -2.7848 |
| Zlat_10016473 | Tricin synthase 1 | -1.9036 |

**Table S4** List of differentially expressed genes in *Ustilago esculenta* under fenaminosulf stress.

| Locus tag | Annotation | log2FoldChange |
| --- | --- | --- |
| KEGG:uma00052 Galactose metabolism  Sample number/Background number:2/13 Corrected P-value: 0.173689177061 | | |
| g813 | Putative invertase | -1.842 |
| g3269 | Alpha-glucosidase | -3.3883 |
| KEGG:uma00650 Butanoate metabolism  Sample number/Background number:2/20 Corrected P-value: 0.183258712357 | | |
| g5895 | Acetoacetyl-CoA synthetase | 3.1926 |
| g352 | 5-aminovalerate aminotransferase | 2.1351 |
| KEGG:uma00500 Starch and sucrose metabolism  Sample number/Background number:2/26 Corrected P-value: 0.192927294722 | | |
| g813 | Putative invertase | -1.842 |
| g3269 | Alpha-glucosidase | -3.3883 |
| KEGG:uma00400 Phenylalanine, tyrosine and tryptophan biosynthesis  Sample number/Background number:1/15 Corrected P-value: 0.371552252837 | | |
| g748 | Phospho-2-dehydro-3-deoxyheptonate aldolase | -100 |
| KEGG:uma00630 Glyoxylate and dicarboxylate metabolism  Sample number/Background number:1/15 Corrected P-value: 0.371552252837 | | |
| g2660 | 2-methylcitrate synthase | -2.3563 |
| KEGG:uma00410 beta-Alanine metabolism  Sample number/Background number:1/16 Corrected P-value: 0.371552252837 | | |
| g352 | 5-aminovalerate aminotransferase | 2.1351 |
| KEGG:uma00640 Propanoate metabolism  Sample number/Background number:1/17 Corrected P-value: 0.371552252837 | | |
| g352 | 5-aminovalerate aminotransferase | 2.1351 |
| KEGG:uma00051 Fructose and mannose metabolism  Sample number/Background number:1/19 Corrected P-value: 0.371552252837 | | |
| g2684 | L-rhamnose-1-dehydrogenase | 2.5426 |
| KEGG:uma00250 Alanine, aspartate and glutamate metabolism  Sample number/Background number:1/22 Corrected P-value: 0.371552252837 | | |
| g352 | 5-aminovalerate aminotransferase | 2.1351 |
| KEGG:uma00020 Citrate cycle (TCA cycle)  Sample number/Background number:1/23 Corrected P-value: 0.371552252837 | | |
| g2660 | 2-methylcitrate synthase | -2.3563 |
| KEGG:uma01230 Biosynthesis of amino acids  Sample number/Background number:2/90 Corrected P-value: 0.371552252837 | | |
| g2660 | 2-methylcitrate synthase | -2.3563 |
| g748 | 2-methylcitrate synthase | -2.3563 |
| KEGG:uma01210 2-Oxocarboxylic acid metabolism  Sample number/Background number:1/25 Corrected P-value: 0.371552252837 | | |
| g2660 | 2-methylcitrate synthase | -2.3563 |
| KEGG:uma04146 Peroxisome  Sample number/Background number:1/39 Corrected P-value: 0.49571460689 | | |
| g6523 | Peroxisomal targeting signal 1 receptor | 2.0429 |
| KEGG:uma04111 Cell cycle - yeast  Sample number/Background number:1/59 Corrected P-value: 0.576271557971 | | |
| g5909 | Structural maintenance of chromosomes protein 2 | 4.4313 |
| KEGG:uma01100 Metabolic pathways  Sample number/Background number:6/555 Corrected P-value: 0.576271557971 | | |
| g980 | 5-hydroxyisourate hydrolase | -5.6573 |
| g352 | 5-aminovalerate aminotransferase | 2.1351 |
| g2660 | 2-methylcitrate synthase | -2.3563 |
| g748 | Phospho-2-dehydro-3-deoxyheptonate aldolase | -100 |
| g813 | Putative invertase | -1.842 |
| g3269 | Alpha-glucosidase | -3.3883 |
| KEGG:uma03013 RNA transport  Sample number/Background number:1/73 Corrected P-value: 0.576271557971 | | |
| g4739 | -//- | 4.133 |
| KEGG:uma00230 Purine metabolism  Sample number/Background number:1/74 Corrected P-value: 0.576271557971 | | |
| g980 | 5-hydroxyisourate hydrolase | -5.6573 |
| KEGG:uma01200 Carbon metabolism  Sample number/Background number:1/78 Corrected P-value: 0.576271557971 | | |
| g2660 | 2-methylcitrate synthase | -2.3563 |
| KEGG:uma03040 Spliceosome  Sample number/Background number:1/80 Corrected P-value: 0.576271557971 | | |
| g2750 | Small nuclear ribonucleoprotein G | -100 |
| KEGG:uma01110 Biosynthesis of secondary metabolites  Sample number/Background number:2/208 Corrected P-value: 0.609968042106 | | |
| g2660 | 2-methylcitrate synthase | -2.3563 |
| g748 | Phospho-2-dehydro-3-deoxyheptonate aldolase | -100 |

**Figure S1**


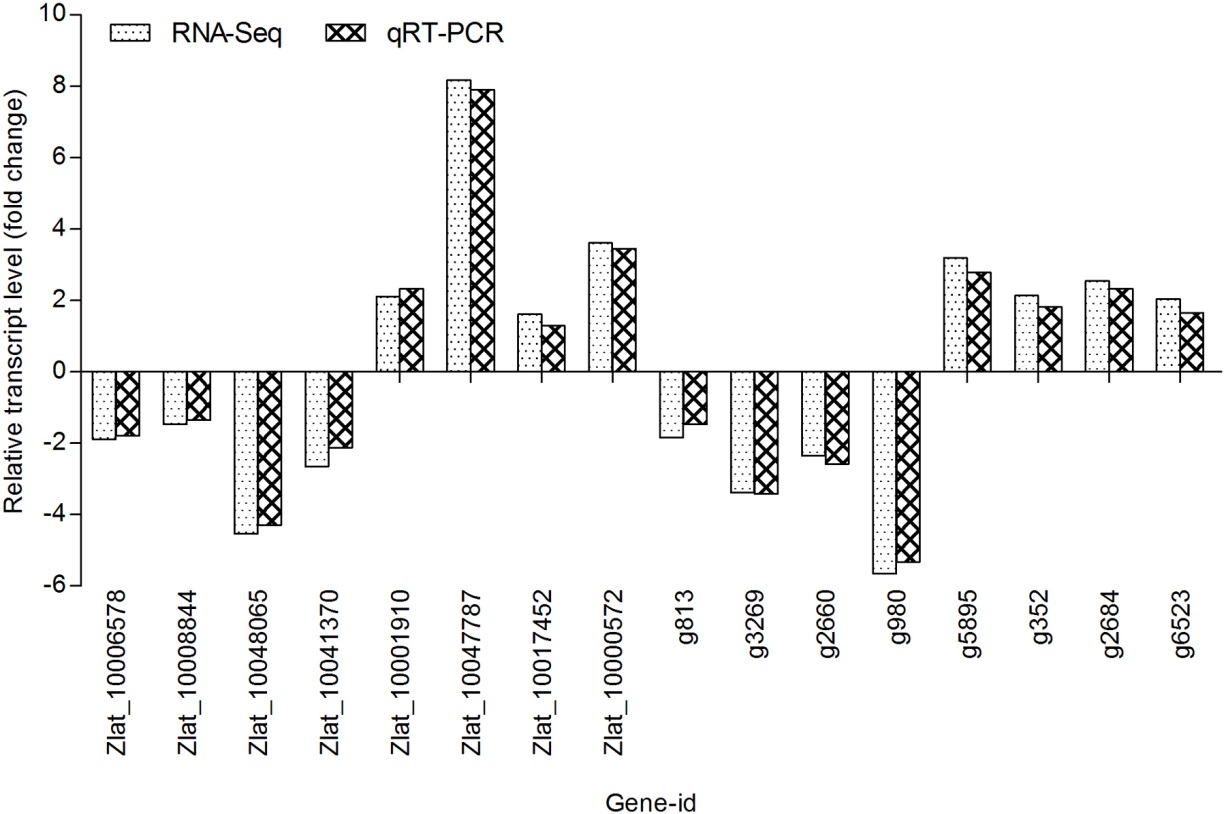


**Figure S1. Comparison of gene expression patterns obtained by RNA-Seq and qRT-PCR.** Changes in relative transcript levels of all 16 genes (8 genes came from *Z. latifolia* and rest from *U. esculenta*) from the same sample were compared by transcriptome and qRT-PCR with paired primers in the Table S2.
